# Blood culture time to positivity in non-β-hemolytic streptococcal bacteremia as a predictor of infective endocarditis—a retrospective cohort study

**DOI:** 10.1007/s10096-021-04339-7

**Published:** 2021-10-16

**Authors:** David Krus, Fredrik Kahn, Bo Nilson, Torgny Sunnerhagen, Magnus Rasmussen

**Affiliations:** 1 Department for Clinical Sciences Lund, Division of Infection Medicine, Medical Faculty, Lund University, BMC B14, Tornavägen 10, 221 84 Lund, Sweden; 2 Division for Infectious Diseases, Skåne University Hospital, Lund, Sweden; 3 Department of Laboratory Medicine Lund, Division of Medical Microbiology, Medical Faculty, Lund University, Lund, Sweden; 4 Department of Clinical Microbiology, Office for Medical Services, Region Skåne, Lund, Sweden

**Keywords:** Time to positivity, Bacteremia, Blood culture, Streptococcus, Infective endocarditis

## Abstract

Non-β-hemolytic streptococci (NBHS) cause infective endocarditis (IE) and a short blood culture time to positivity (TTP) is associated with risk of IE in bacteremia with other pathogens. In this retrospective population-based cohort study, we investigate if TTP is associated to IE or mortality. Of 263 episodes with NBHS bacteremia, 28 represented IE and the median TTP did not differ significantly between episodes with IE (15 h) and non-IE (15 h) (*p*=0.51). TTP was similar among those who survived and those who died within 30 days. However, TTP significantly differed when comparing the different streptococcal groups (*p*<0.001).

**Supplementary Information:**

The online version contains supplementary material available at 10.1007/s10096-021-04339-7.

## Introduction

Streptococcal bloodstream infections are complicated by infective endocarditis (IE) in approximately 7% of cases [1]. Non-beta-hemolytic streptococci (NBHS) are common causative agents of IE [1, 2]. Especially the *Streptococcus mutans* group, the *Streptococcus sanguinis* group and the *Streptococcus bovis* group are associated with IE. In NBHS bacteremia heart valve disease, long duration of symptoms and community acquisition are also associated with increased risk of IE [1, 3].

Blood culture time to positivity (TTP) provides indirect information on the bacterial concentration in blood. However, TTP is affected by other factors such as the microbial growth rate and logistics around the blood sampling and transportation. Since IE is an intravascular infection, it has been speculated that high numbers of bacteria are present in the blood, and indeed, cases with *Staphylococcus aureus* or *Enterococcus faecalis* IE have shorter TTP compared to cases of bacteremia with these pathogens without IE [4–7] [8].

This study aims to examine whether TTP in NBHS bacteremia is associated with IE. Moreover, we aimed to examine if TTP in NBHS bacteremia is associated with mortality or other clinical and microbiological variables.

## Methods

This is a retrospective observational study, comprising a cohort of 481 episodes of NBHS bacteremia in the province of Skåne, Sweden, from 1 January 2015 through 31 March 2016. A dataset of all patients with NBHS-bacteremia were obtained from the Laboratory for Clinical Microbiology and were reviewed according to a predefined protocol (Supplemental data). Patients from the five largest hospitals were included and these all had BACTEC FX blood culture systems (Becton Dickinson, Franklin Lakes, USA) where bottles were inserted directly (24/7). Positive bottles were transferred to the central laboratory and species determination was performed using MALDI-TOF MS (MALDI Biotyper Compass 4.1, with MBT Compass Library, DB-6903 MSP (Bruker, Bremen, Germany)). The NBHS were categorized into six groups; the *S. bovis* group, the *S. sanguinis* group, the *S. salivarius* group, the *S. mitis* group, the *S. anginosus* group, and the *S. mutans* group [3]. The study was approved by the Regional Ethics Committee (Reg nr. 2013/31).

Patients were excluded if all cultures were polymicrobial, age <18 years, and if patient records were unavailable. TTP was defined by the shortest TTP in the bottles with monomicrobial growth of NBHS from a given patient. The diagnosis of IE was based on the modified Duke criteria [9, 10] and only episodes fulfilling the definite criteria were regarded as IE. Comorbidities were graded using the Charlson comorbidity index [11]. Neutropenia was defined as absolute neutrophil count < 0.5 × 10^9^/L [3]. Consideration was given to infection site of acquisition, i.e., if infection was community, health care or nosocomial acquired [12]. The focus infection was defined according to Berge et al. [13] (for details, see Supplementary Data).

Statistics were based on NBHS-episodes (*n*=263) rather than number of patients (*n*=253). Patients with confirmed IE or absence of IE were compared using *χ*^2^-test, Fisher’s exact test, or Mann-Whitney*U* test. Kruskal-Wallis test was performed comparing TTP for individual species within the NBHS group. Multivariable linear regression analysis was performed analyzing TTP in relation to clinical characteristics. Because of a skewness of TTP (Supplemental Figure 1A) and the corresponding residuals for the regression (Supplemental Figure 2A), the assumptions for linear regression were not met. Hence, TTP was log-transformed(Supplemental Figure 1B and 2B). A multivariable regression was performed with ln TTP as outcome. The beta coefficients were then back-transformed to the linear scale. Collinearity was assessed by variance inflation factor (VIF)(Supplemental Table 1). Multivariable analysis and subsequent analysis were performed in R Foundation for Statistical Computing, Vienna, Austria version 3.6.2 with the packages haven, janitor, tidyr, broom, ggplot2, and car.

## Results

### Description of the cohort

From a total of 481 episodes with NBHS bacteremia, 218 episodes were excluded; 35 episodes due to the fact that the hospital did not hold a blood culture incubator, 153 due to exclusively polymicrobial cultures, 57 due to age <18 years, and 4 due to unobtainable records. Thus, there were 263 episodes in 253 patients that met the inclusion criteria of which 28 (11%) fulfilled modified Duke criteria for definite IE. Sixty-three percent of patients had undergone any echocardiography and 35% had been subjected to transesophageal echocardiography. In Table 1, a description of the cases with IE and non-IE is given.

**Table 1:** Clinical characteristics and outcome in cases of NBHS bacteremia in relation to IE

Characteristic	All cases (*n*=263)	IE (*n*=28)	Non-IE (*n*=235)	*p* value, IE vs. non-IE
Age, median (IQR)	71 (59-81)	73 (63-82)	71 (59-81)	0.34
Sex				0.025
Female	108 (41)	6 (21)	102 (43)	
Male	155 (59)	22 (79)	133 (57)	
Charlson score, median (IQR)	2 (0–3)	0 (0–1)	2 (0–3)	<0.001
Site of acquisition				<0.001
Community	146 (56)	25 (89)	121 (51)	
Non-community	117 (44)	3 (11)	114 (49)	
TTP, median hours (IQR)	15 (12–21)	15 (12–19)	15 (12–21)	0.51
Echocardiography performed (TTE or TEE)	167 (63)	28 (100)	139 (59)	< 0.00001
TEE performed	93 (35)	26 (93)	67 (29)	< 0.00001
NBHS group				1
*S. bovis*	19 (7.2)	1 (3.6)	18 (7.7)	
*S. mutans*	7 (2.7)	3 (11)	4 (1.7)	
*S. sanguinis*	32 (12)	7 (25)	25 (11)	
*S. mitis*	105 (40)	12 (43)	93 (40)	
*S. salivarius*	17 (6.5)	3 (11)	14 (6.0)	
*S. anginosus*	83 (32)	2 (7.1)	81 (35)	
Neutropenia^2^				0.034
Yes	35 (13)	0 (0.0)	35 (15)	
No	228 (87)	28 (100)	200 (85)	
Other focal infection				0.24
Yes^3^	61 (23)	4 (14)	57 (24)	
No	202 (77)	24 (86)	178 (76)	
Death within 30 days^4^				0.088
Yes	24 (9.1)	0 (0.0)	24 (10)	
No	239 (91)	28 (100)	211 (90)	

Data is presented as numbers with percentages within parenthesis unless otherwise indicated. Statistical analyses were performed using Mann-Whitney*U* test or Fisher’s exact test. *IQR*, inter quartile range; *TTE*, transthoracic echocardiography; *TEE*, treansesophageal echocardiography; *TTP*, time to positivity. ^1^*p* value not possible to calculate due to few observations in some subgroups. ^2^Absolute neutrophil count < 0.5 × 10^9^/L at the time positive blood culture was drawn. ^3^Including osteomyelitis, spondylodiscitis, gastroinstestinal infection, urogenital infection, airway infection, and soft tissue infection. ^4^From the time positive blood culture was drawn

### Time to positivity and IE

TTP was compared between IE- and non-IE-episodes. Median TTP for IE episodes was 15 h (IQR 12–19 h) and non-IE 15 h (IQR 12–21 h) (*p*=0.51 for difference) (Table 1). There was no association between TTP and IE in the univariate analysis and there were few IE cases we, therefore, chose not to perform multivariate regression with IE as outcome.

As sensitivity analyses, we also compared TTP between patients with definite IE with those with IE rejected (excluding patients with possible IE), between patients treated as IE and those not treated as IE (irrespective of Dukes criteria), and only among persons who underwent TEE (Supplemental Figure 3). There were no significant differences in TTP between any of these groups.

### Time to positivity in relation to mortality

The median TTP was 17 h (IQR 12–24) in episodes where the patient died within 30 days (*n*=24) compared to 15 h (IQR 12–21) in the 239 episodes where the patient survived (*p*=0.37 for difference) (data not shown). There were no significant differences in TTP between survivors and non-survivors at hospital discharge or at 6 months either.

### Other features possibly related to TTP

As a secondary analysis, we investigated if other features such as clinical and bacteriological variables were associated with a particular TTP as the outcome. Significant differences were observed in five characteristics: sex, site of acquisition, NBHS group, other focal infection and neutropenia. However, after multivariate regression analysis, the only variable revealing statistical significance was streptococcal group where the *S. mutans* and the *S. anginosus group* had a median TTP of approximately 20 h compared to around 10 h for *S. bovis* and *S. salivarius*(Table 2).

**Table 2:** Clinical characteristics NBHS bacteremia episodes in relation to TTP

Characteristic	Episodes	TTP, median	TTP, mean	*p* value univariate^3^	*p* value multivariate^4^	Multiplication vs. reference level (exponent of beta)
All episodes	263 (100)	15 (12–21)	18			
Age (years)				0.080	0.92	0.99
18–70	124 (47)	14 (12–20)	18			
≥ 71	139 (53)	16 (13–22)	18			
Sex				0.044	0.15	0.93
Male	155 (59)	16 (12–22)	19			
Female	108 (41)	15 (12–19)	17			
Charlson score				0.12	0.80	1.01
0–1	130 (49)	16 (13–22)	19			
≥ 2	133 (51)	15 (12–21)	18			
Site of acquisition				0.006	0.53	0.96
Community	146 (56)	17 (13–23)	20			
Non-community	117 (44)	14 (12–20)	17			
Infective endocarditis				0.51	0.54	0.95
Yes	28 (11)	15 (12–19)	17			
No	235 (89)	15 (12–21)	18			
NBHS group				< 0.001		
*S. bovis*	19 (7.2)	10 (8.8–13)	12		0.006	0.75
*S. mutans*	7 (2.7)	22 (20–28)	26		0.002	1.66
*S. sanguinis*	32 (12)	16 (12–19)	18		0.27	1.10
*S. mitis*	105 (40)	14 (12–17)	15		5	
*S. salivarius*	17 (6.5)	12 (11–13)	13		0.09	0.83
*S. anginosus*	83 (32)	21 (17–26)	24		<0.001	1.45
Neutropenia^1^				< 0.001	0.15	0.88
Yes	35 (13)	13 (11–16)	14			
No	228 (87)	16 (13–22)	19			
Other focal infection^2^				0.005	0.91	0.99
Yes	61 (23)	18 (13–24)	22			
No	202 (77)	15 (12–20)	17			

“All cases” are presented as numbers (%). Time to positivity (TTP) is presented as median hours with interquartile range (IQR). ^1^Absolute neutrophil count < 0.5 × 10^9^/L at the time positive blood culture was drawn. ^2^Including osteomyelitis, spondylodiscitis, gastroinstestinal infection, urogenital infection, airway infection, and soft tissue infection. ^3^Univariate statistical analyses were performed using the Mann-Whitney*U* test or the Kruskal-Wallis test. ^4^Statistical analysis performed via multivariate regression analysis. ^5^*S. mitis* indicated as baseline NBHS group in statistical analysis

## Discussion

We and others have observed a strong association between a short TTP and IE in *S. aureus* [6] [4] [5] and *E. faecalis* bacteremia [8], indicating that if present, such differences would be feasible to identify also for NHBS. NBHS are, in contrast to *S. aureus* and *E. faecalis*, a heterogenous entity comprised of many different species with different properties and propensities to cause IE [1, 3]. Not all that surprisingly, there were clear differences when comparing TTP between different groups within NBHS. These differences can be due to growth characteristics of isolates. However, we cannot exclude that they could also reflect the biological properties of the types of infections that the different NHBS groups cause despite that we tried to control for this in the multivariable analysis.

This study indicates that TTP in NBHS bacteremia neither correlate with IE as defined by the Duke criteria nor with mortality and this is clearly different from the situation in *S. aureus* bacteremia. A reason for the NHBS bacteremia episodes with IE not displaying a shorter TTP could be that only few bacteria from the vegetation are released into the blood of the patient. However, it might also reflect that NBHS from an IE vegetation have a slower growth rate since it has been shown that streptococci inside vegetation with time develop a state of low metabolic activity [14].

Weaknesses of this study due to the retrospective design include risk of misclassifying IE episodes as non-IE due to underuse of echocardiography, a lack of information on time from blood culture to start of incubation, and on the actual blood volumes in culture flasks. Moreover, despite being relatively large, the number of episodes with a given NHBS group is low hampering the possibility to detect small differences in subgroups. A strength of the study is that all cultures were performed in hospitals having blood culture cabinets and thus similar times from the drawing of the blood until they enter the blood culture cabinet. This will likely decrease variability in TTP and would facilitate the detection of true differences.

In conclusion, TTP varies between different subgroups of NBHS but it does not appear to provide clinically meaningful information in patients with NBHS bacteremia.

## Supplementary Information

ESM 1 (PNG 389 kb)

High resolution image (TIF 5380 kb)

ESM 2 (PNG 3852 kb)

High resolution image (TIF 5381 kb)

ESM 3 (PNG 531 kb)

High resolution image (TIFF 1605 kb)

ESM 4 (DOCX 44 kb)

## Data Availability

Pseudonymized data will be made available upon reasonable request.
