# Supplementary figures and images for: Blood culture time to positivity in non-β-hemolytic streptococcal bacteremia as a predictor of infective endocarditis—a retrospective cohort study
Source: Eur J Clin Microbiol Infect Dis. 2021 Oct 16;41(2):325–9. doi: 10.1007/s10096-021-04339-7 (PMC8770443; doi:10.1007/s10096-021-04339-7)

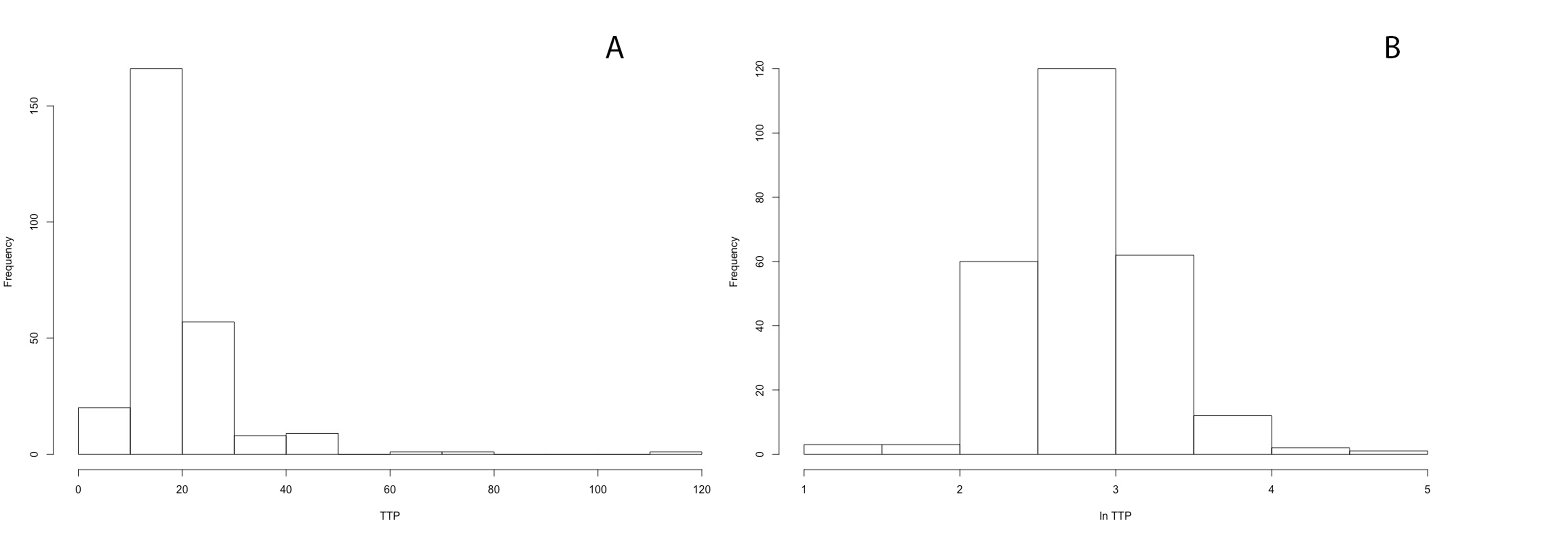

Supplement: Supplementary file 1 — (PNG 389 kb) [file 10096_2021_4339_Fig1_ESM.png]

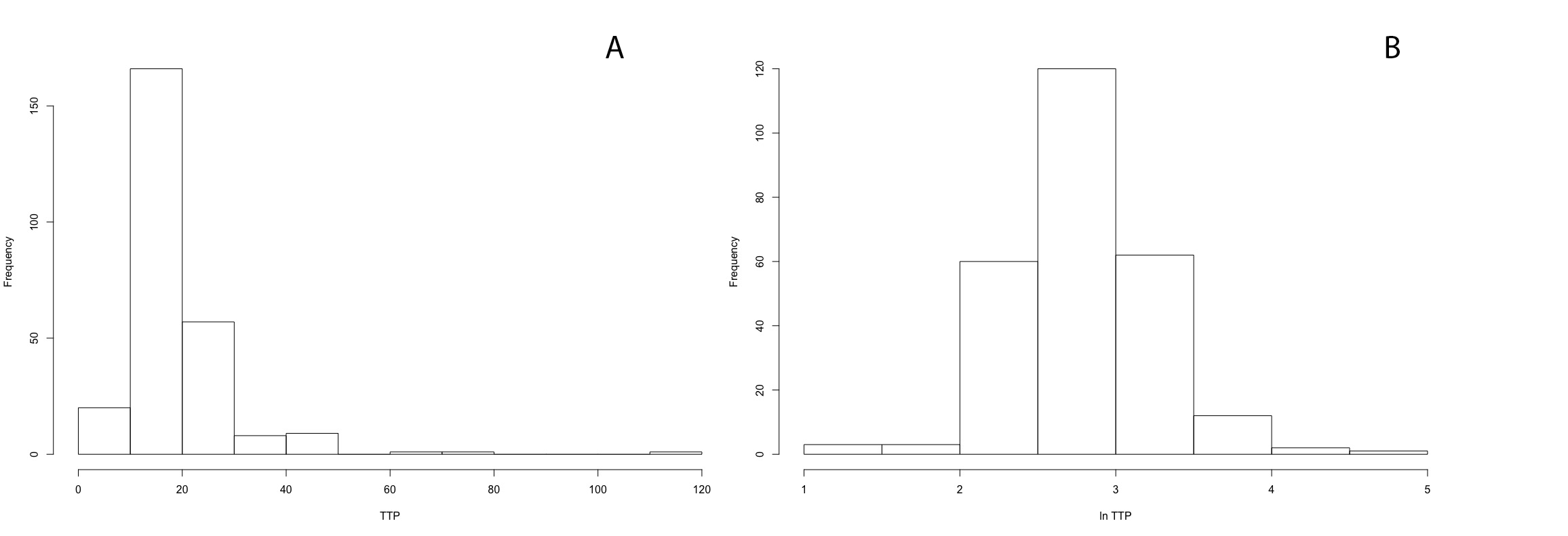

Supplement: Supplementary file 2 — High resolution image (TIF 5380 kb) [file 10096_2021_4339_MOESM1_ESM.tif]

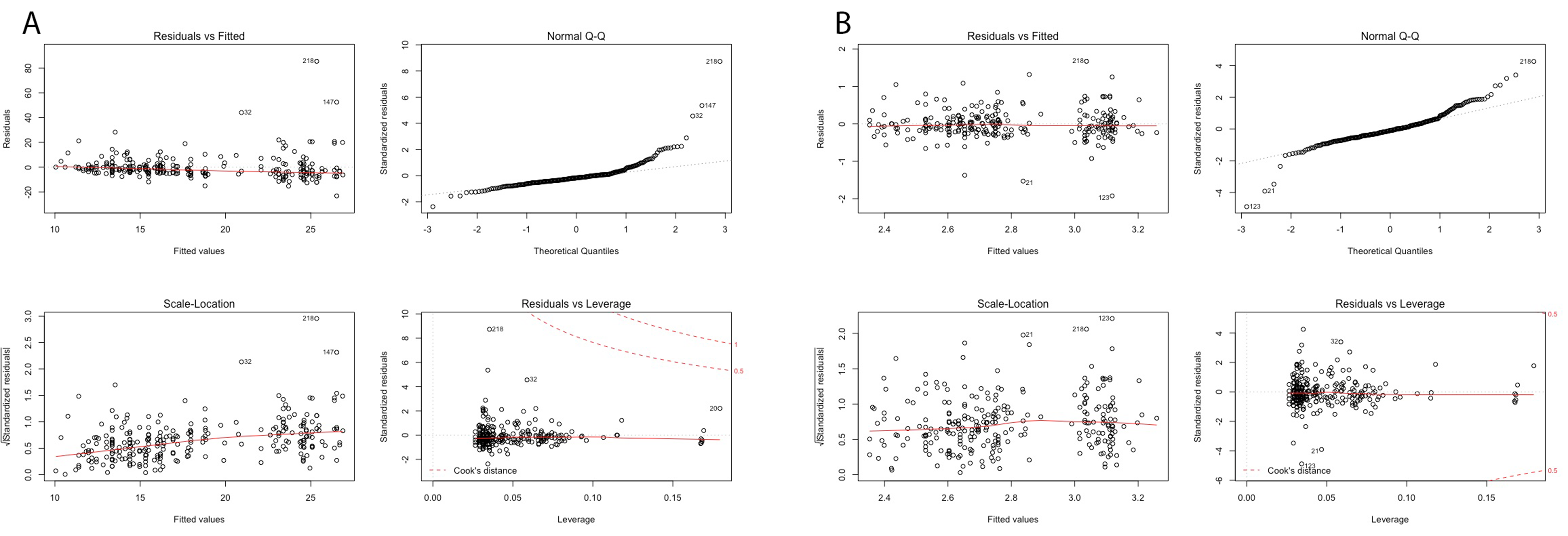

Supplement: Supplementary file 3 — (PNG 3852 kb) [file 10096_2021_4339_Fig2_ESM.png]

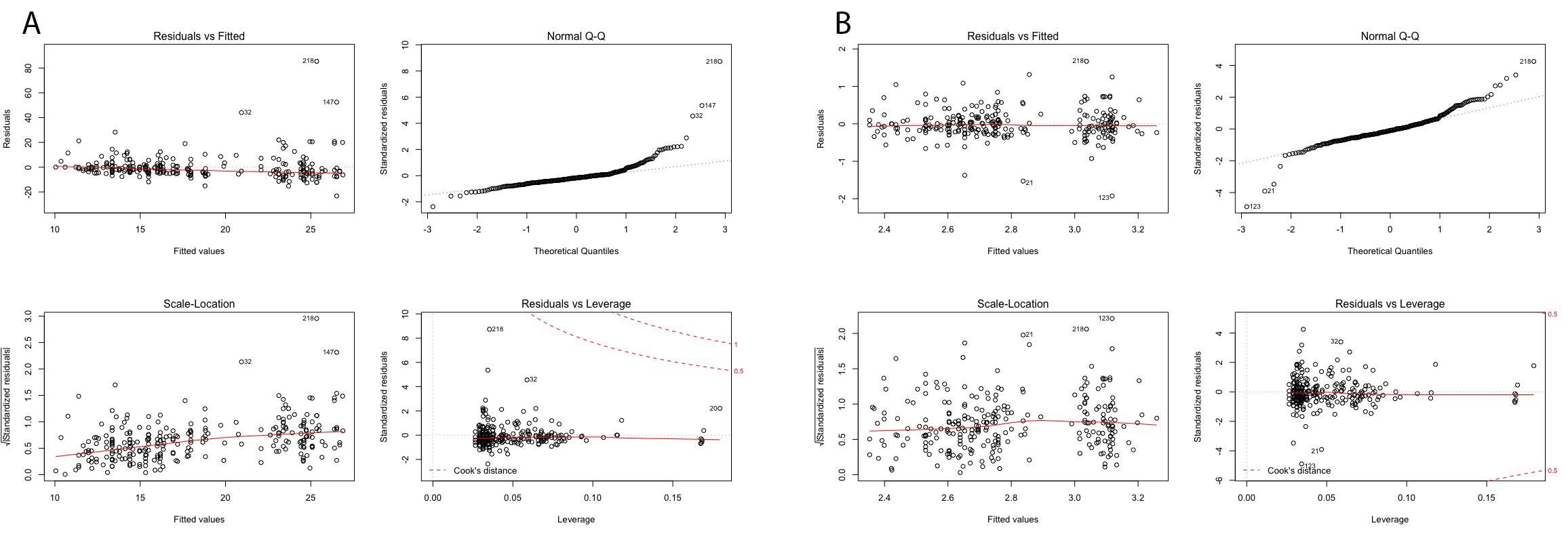

Supplement: Supplementary file 4 — High resolution image (TIF 5381 kb) [file 10096_2021_4339_MOESM2_ESM.tif]

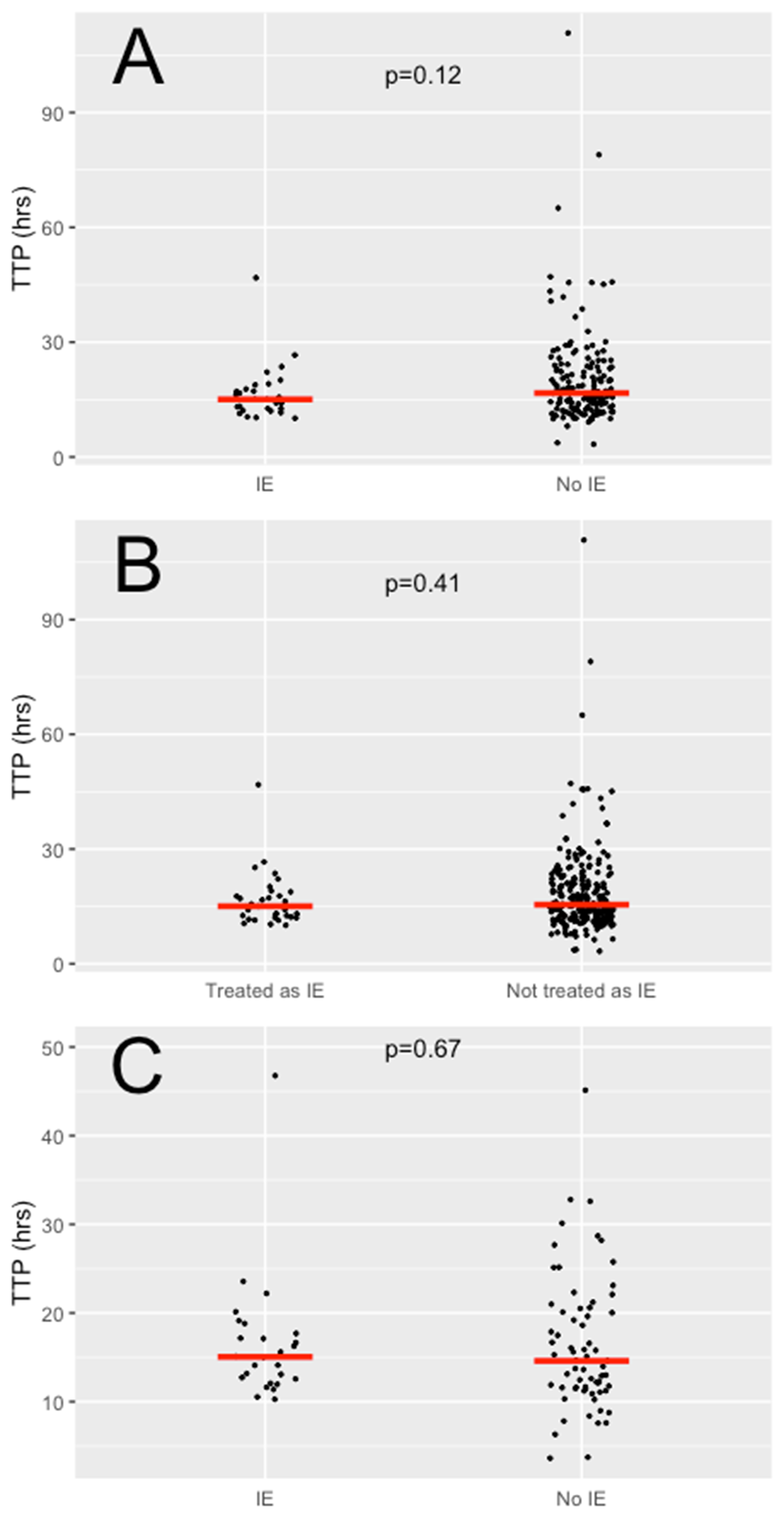

Supplement: Supplementary file 5 — (PNG 531 kb) [file 10096_2021_4339_Fig3_ESM.png]

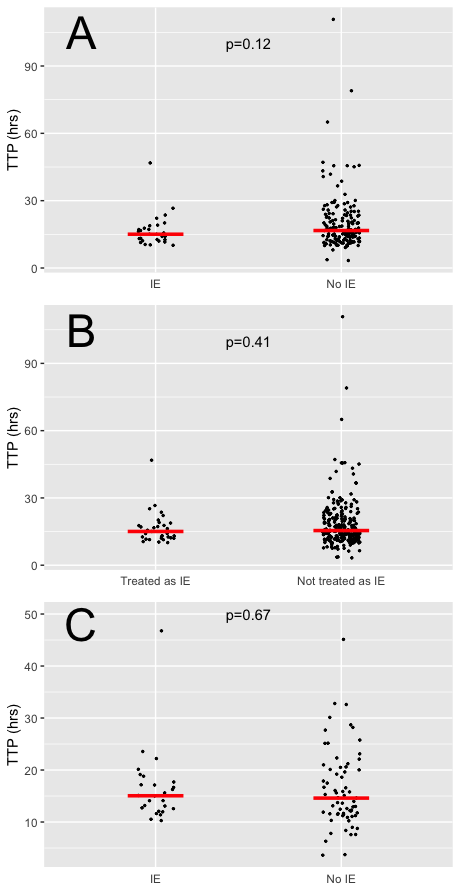

Supplement: Supplementary file 6 — High resolution image (TIFF 1605 kb) [file 10096_2021_4339_MOESM3_ESM.tiff]
