# Supplementary material for: Blood culture time to positivity in non-β-hemolytic streptococcal bacteremia as a predictor of infective endocarditis—a retrospective cohort study
Source: Eur J Clin Microbiol Infect Dis. 2021 Oct 16;41(2):325–9. doi: 10.1007/s10096-021-04339-7 (PMC8770443; doi:10.1007/s10096-021-04339-7)
Supplement: Supplementary file 7 — (DOCX 44 kb) [file 10096_2021_4339_MOESM4_ESM.docx]

# Supplementary data

**Supplementary text**

The parameters included in the protocol were: age, sex, Charlson score [11], HANDOC score [3], site of acquisition – nosocomial, community or health care-associated, modified Duke criteria [10], pacemaker/ICD, TTE performed, TTE with signs of IE, TEE performed, TEE with signs of IE, PET-CT performed, PET-CT with signs of IE, ECG-triggered CT performed, ECG triggered CT with signs of IE, death in hospital, death within 30 days of hospital admission, death within six months of hospital admission, days in hospital, admitted intensive care unit during hospital stay, heart surgery, relapse within 180 days, focal infection [13], days of antibiotic treatment.

Community acquired bloodstream infection was defined by detected bacteremia from blood cultures drawn within 48 hours from hospital admission for patients who did not meet the criteria for health care-associated bloodstream infection. Health care associated bloodstream infection was defined as community acquired if the patient also fulfilled the following criteria: (1) Received intravenous therapy at home or received wound care or specialized nursing care in the 30 days before the bloodstream infection; (2) attended a hospital or hemodialysis clinic or received chemotherapy in the 30 days before the bloodstream infection; (3) was hospitalized in an acute care hospital for 2 or more days in the 90 days before the bloodstream infection; (4) resided in a nursing home or long-term care facility. Nosocomial infection was defined by bacteremia from blood cultures drawn more than 48 hours from hospital admission [12].

In accordance with the work on DENOVA, focal infection was defined as fulfillment of two of the following criteria; (1) typical signs or symptoms of infection, (2) isolation of the same

NBHS at the site of infection as in bloodstream, (3) imaging results compatible with focal infection which was likely to be the point of entry of the bacteria [13].

**Supplemental Table 1.** Variance inflation factor (VIF) was assessed using the car package from R.

|  | **GVIF** | **Df** | **GVIF^(1/(2*Df))** |
| --- | --- | --- | --- |
| Age | 1.131703 | 1 | 1.063815 |
| Sex | 1.051061 | 1 | 1.025212 |
| Charlson | 1.342382 | 1 | 1.158612 |
| Site of acquisition | 1.395030 | 1 | 1.181114 |
| Endocarditis | 1.220212 | 1 | 1.104632 |
| Other focal infection | 1.250953 | 1 | 1.118460 |
| Neutropenia | 1.408627 | 1 | 1.186856 |
| Streptococcal groups | 1.570268 | 5 | 1.046158 |

**Legends to supplemental figures**

**Supplemental figure 1.** Histogram of a) TTP and b) ln TTP.

**Supplemental figure 2.** Assessment of the assumptions for linear regression for a) TTP and b) ln TTP. Each of the four panels depict the residuals vs. fitted values, a normal Q-Q plot of the residuals, a scale location plot and, a residuals vs. leverage plot.

**Supplemental figure 3.** Sensitivity analyses of relation between IE and TTP. In A) a comparison of TTP between cases with IE (definite IE according to Duke criteria) (n=28) and no IE (Duke IE rejected and not treated as IE) (n=157), thus excluding cases with possible IE and persons treated as IE despite not fulfilling definite criteria for IE. Median TTP was 15 hrs for the IE group and 16.7 hrs for the non-IE group, this difference was not statistically significant (p= 0.12 with Mann Whitney-U test) In B) a comparison between cases treated as IE irrespective of fulfilling Dukes criteria (n=34) and not treated as IE (n=234). Median TTP was 15 hrs for the “treated as IE” group and 15.4 hrs for the “not treated as IE group”, this difference was not statistically significant (p= 0.41 with Mann Whitney-U test). In C) a comparison between patients who have undergone investigation with transesophageal echocardiography (TEE) (n=93) with definite IE (n=26) or not definite IE (n=67). Median TTP was 15 hrs for the IE group and 14.6 hrs for the non-IE group, this difference was not statistically significant (p= 0.67 with Mann Whitney-U test).
